# Climate change impacts on crop yield, soil water balance and nitrate leaching in the semiarid and humid regions of Canada

**DOI:** 10.1371/journal.pone.0207370

**Published:** 2018-11-16

**Authors:** Wentian He, J. Y. Yang, B. Qian, C. F. Drury, G. Hoogenboom, P. He, D. Lapen, W. Zhou

**Affiliations:** 1 Institute of Agricultural Resources and Regional Planning, Chinese Academy of Agricultural Sciences (CAAS), Beijing, China; 2 Harrow Research and Development Centre, Agriculture and Agri-Food Canada, Harrow, ON, Canada; 3 Ottawa Research and Development Centre, Agriculture and Agri-Food Canada, Ottawa, Canada; 4 Institute for Sustainable Food Systems, University of Florida, Gainesville, Florida, United States of America; Universiteit Utrecht, NETHERLANDS

## Abstract

The impact of climate change on agricultural systems is a major concern as it can have a significant effect on the world food supply. The objective of this study was to evaluate climate change impacts on crop production and nitrate leaching in two distinct climatic zones in Canada. Spring wheat (*Triticum aestivum* L.) was selected for the semiarid regions of Western Canada (Swift Current, SK) and maize (*Zea mays* L.) was chosen for the more humid regions of central Canada (Woodslee, ON). Climate scenarios were based upon simulations from a Canadian Regional Climate Model (CanRCM4) under two Representative Concentration Pathways (RCP4.5 and RCP8.5) and crop simulations were conducted using the Decision Support System for Agrotechnology Transfer (DSSAT) model. Compared to the baseline climate scenario, wheat yields increased by 8, 8, 11, 15%, whereas maize yields decreased by 15, 25, 22, 41% under RCP4.5 2050s (2041–2070), RCP4.5 2080s (2071–2100), RCP8.5 2050s and RCP8.5 2080s scenarios, respectively. Annual nitrate leaching increased by 19, 57, 73, 129% at Swift Current and by 84, 117, 208, 317% at Woodslee under the four scenarios, respectively. Adaptation measures suggested that fertilizer N rate for spring wheat should be increased to 80–100 kg N ha^-1^ to obtain optimal yields although this will result in an additional risk of 5–8 kg N ha^-1^ nitrate leaching at Swift Current. The fertilizer N rate of 150 kg N ha^-1^ was found to be suitable for high maize yields at Woodslee. New wheat and maize cultivars with long growing seasons would enable crop growth to match the phenological stage and hence maintain high crop yields to adapt to increased temperatures in the future.

## Introduction

Greenhouse gas (GHG) emissions have increased markedly and their atmospheric concentrations will continue to rise during the 21^st^ century unless mitigation measures are adopted [1]. The GHG emissions increased by about 70% from 1970–2004 and carbon dioxide (CO_2_) alone increased by about 80% [2]. The evidence for rapid climate change is compelling, including the increase of air temperatures, the rise of sea levels, the change of precipitation patterns and the increasing frequency of extreme weather events [3]. Agricultural crop production could be significantly affected by change in atmospheric CO_2_ concentration and the accompanying climate change due to differences in photosynthesis, crop respiration, water use efficiency (WUE) as well as soil biological and chemical transformations of C and N [4–6]. The positive response to elevated CO_2_ concentration might be reduced by an increased temperature, increased evapotranspiration or drought stress. Therefore, climate change may have varying effects on crop production depending on soil, management and climatic conditions [3,7,8].

Process-based simulation models have been widely used in agricultural research for developing cropping technologies, exploring management practices and assessing policy decisions [9,10]. Agricultural system models can simulate crop growth, soil water balance and nutrient cycling in daily time steps [11–14]. They are applied to evaluate the impacts of agricultural management practices on crop yield and nutrient dynamics under different climatic conditions [15,16]; aid the development of tools for farmers or policy applications [17,18]; and assess the effects of climate change on crop production, environment risks as well as explore potential adaptation measures [19–21].

Spring wheat (*Triticum aestivum* L.) accounts for the largest cropping area (14.8%) in Canada and 96% of the total wheat is grown in the semiarid regions of Western Canada [22,23]. Maize (*Zea mays* L.) is one of the most important cereal crops in Central Canada (Ontario and Quebec), and about 90–94% of Canadian maize production occurs in these two provinces (Yang *et al*. 2013). Canada plays an important role in global food supply and any changes in wheat and maize production in Canada due to climate change will impact the global wheat and maize market [24,25].

Global fertilizer N application has greatly contributed to increased food and feed production since the early 20^th^ century. However, substantial amounts of fertilizer nitrogen could be lost from agricultural systems to the atmosphere through volatilization (NH_3_) and denitrification (e.g., N_2_O, N_2_), and to rivers and lakes through leaching or runoff (NO_3_) which in turn contributes to low nitrogen use efficiency by crops[26–29]. For example, N is the primary cause of eutrophication in many coastal ecosystems [30]. In Australia, it was reported that nitrate in approximately half the wells with elevated N concentrations was likely to have originated from fertilizer [31].

Integrated assessments of N losses have been conducted to evaluate serious N leakage (e.g., NO_3_, N_2_O, NH_3_ etc.) from agricultural systems to the environment since the 1990s in the EU countries [28]. Although a series of environmental policies were adopted and contributed to decreasing N losses, the nitrate concentrations in groundwater and surface waters were still high, which threatened human health and water quality. In Canada, modelling studies on residual soil nitrogen (RSN) and nitrate leaching in farmland from 1981 to 2011 have been conducted [32,33]. Annual RSN levels (i.e., annual RSN = N input–N output) increased from 9.4 kg N ha^-1^ in 1981 to 23.6 kg N ha^-1^ in 2011 in Canadian farmland. However, NO_3_ leaching loss varied from 3–14% of the RSN in the semi-arid regions of Western Canada whereas 33–90% of RSN was lost in the more humid regions in Eastern Canada as a result of different climatic conditions, soil types and cropping systems [34].

The predicted changes in temperature, precipitation patterns and CO_2_ concentrations in the future could significantly impact the fluxes of soil mineral N and N leaching [35]. Patil et al. [36] modelled the impact of changes in climatic variables on both winter wheat yield and nitrate leaching from soils in Denmark, and indicated that N leaching increased with temperature, particularly for coarse, sandy soils compared to the sandy, loam soils. Similarly, Wang et al. [20] reported that soil nitrate leaching in tile drainage under future climate conditions increased by 34% for a corn-soybean rotation cropping system compared to the historical climate in Iowa, USA, which was attributed to the reduced corn N uptake. Modelling studies have examined whether management practices (crop rotation, tillage and fertilizer optimization), could reduce N leaching and increase crop yields under future climatic conditions. For example, Biggs et al. [37] found that optimizing fertilizer and tillage management practices would be important to reduce N loss with deep drainage under future climate change scenarios. Chauhan et al. [38] predicted that a rotation including wet-season peanut and dry-season maize could result in greater yields and reduced N losses compared to the conventional rotation with wet-season maize and dry-season peanut under climate projections in Australia.

In semiarid regions of Canada, nitrate leaching from cropland can still take place and impact water quality depending on the timing of the precipitation events in relationship to nitrogen application and crop uptake [39]. In Southwestern Ontario, high NO_3_ concentrations from N leaching were found in surface waters due to intensive maize cropping with high N inputs and humid climatic conditions [33,40]. A number of modelling studies were carried out to simulate N losses and crop yields in different crop management systems. However, studies in these two different climatic zones have focused on assessing the effects of future climate change scenarios on crop growth and N_2_O emissions [19,25,41]. Therefore, evaluating the impacts of climate change on soil nitrogen leaching and exploring potential adaptation measures to reduce leaching while increasing crop yield are essential to supporting policy decisions for food security. The main objectives of this study were to: (1) simulate the potential impacts of climate change on spring wheat and maize yields, soil water balance and nitrate leaching; (2) explore adaptation measures to obtain high crop yields and low nitrate leaching losses under different climate change scenarios in the semiarid regions of the Western Canada (Swift Current, Saskatchewan) and the more humid regions of Eastern Canada (Woodslee, Ontario).

## Materials and methods

### Study area

Two sites were selected in Western and Eastern Canada to include diverse climate conditions and cropping systems at the start of this study. The first site was a spring wheat experiment conducted at Swift Current, Saskatchewan (50°170’N, 107°480’W) in the Canadian semiarid temperate climatic zone. The average annual precipitation and temperature are 351 mm and 3.9°C respectively. The soil is an Orthic Brown Chernozem and the soil texture (0–0.15 m) is 50% silt and 20% clay [42]. The second site was a long-term maize cropping system in the Canadian humid-temperate climate at Woodslee, Ontario (42°13’N, 82°44’W) with an average annual precipitation of 843 mm and an average annual temperature of 9.3°C. The soil is a Brookston clay loam with an average soil texture (0–0.15m) of 35% silt and 37% clay [43].

### Climate scenarios

Future climate scenarios used in this study were based on climate change simulations by CanRCM4 [44], a new regional climate model developed at the Canadian Centre for Climate Modelling and Analysis (CCCma) of Environment Canada. CanRCM4 was driven by the global climate model CanESM2 [45] using climatic forcing scenarios Representative Concentration Pathway (RCP) 4.5 and RCP 8.5, which are medium-low and high emission scenarios with a radiative forcing of 4.5 and 8.5 w/m^2^ at the end of the 21^st^ century [8], respectively. Daily outputs from CanRCM4 were obtained from the CCCma website (http://www.cccma.ec.gc.ca/data/data.shtml) for the historical baseline period 1971–2000 and for two future periods of 2041–2070 and 2071–2100, hereafter referred to as the “2050s” and “2080s”. In this study, a bias correction method described in SI (Supporting Information) was applied to the CanRCM4 outputs to develop the climate scenarios data to drive the Decision Support System for Agrotechnology Transfer (DSSAT) model.

### DSSAT model

The DSSAT model has been widely used to simulate crop growth, soil water balance and soil C and N dynamics under different crop systems, management practices and various climatic conditions [11,46–49]. It has also been used to support crop management policy decisions and to simulate the effects of climate change on crop production [50,51]. The DSSAT v4.6 (http://dssat.net/) is integrated with widely used Cropping System Models (CSM), a soil water balance module, and two soil nitrogen and organic matter modules (the CERES-based and CENTURY-based soil models) [50]. The CSM can simulate the growth of 42 different crops as well as for fallow fields. The CENTURY-based soil module was found more suitable for long-term sequence simulations [52], and it was selected to simulate soil nitrogen and carbon dynamics in our research. The soil water balance module is based on the Ritchie equation to calculate daily soil water changes [9]. In this study, the DSSAT Sequence program was used to simulate multi-year soil and water dynamics in addition to crop growth processes. This program is designed to analyze the long term effects of management practices or climate change on crop growth, soil water movement, soil carbon and nitrogen processes. Initial soil conditions are only required to be set up before the first year simulation, then soil water, carbon and nitrogen flows are transferred to the next season automatically [49,53]. In this study, the calibrated DSSAT model was used to explore potential impacts of climate change on spring wheat and maize yields, soil water balance and nitrate leaching. Detailed model input parameters and calibration were introduced in SI (Tables A and B in S1 File).

#### Model runs and analysis

One baseline and four climate scenarios were generated and used for the modelling study, including (1) baseline 1971–2000; (2) RCP4.5 2050s; (3) RCP4.5 2080s; (4) RCP8.5 2050s and (5) RCP8.5 2080s. In order to investigate the impact of individual climatic variables from projected CO_2_, temperature and precipitation, each future scenario was subdivided into four sub-scenarios: (a) using future CO_2_ while keeping other climate variables at baseline values; (b) using future temperature while keeping other climate variables at baseline values; (c) using future precipitation while keeping other climate variables at baseline values; (d) using all variables from future scenarios. Therefore, the CSM-CERES-Wheat and -Maize models in DSSAT were run 1020 times (1 baseline and 4 scenarios by 4 sub-scenarios in a 30-years period) for climate scenario analysis at Swift Current, Saskatchewan and Woodslee, Ontario (Table C-a in S1 File). Four outputs were analysed in this study, including crop yield at harvest, soil water balance components, daily soil mineral N in the profile and daily soil NO_3_ leaching below the soil profile (1.2 m).

The CSM-CERES-Wheat and CSM-CERES-Maize models were driven by the climate input under different climate scenarios. The results from different scenarios were expressed as percentage changes with respect to the baseline and as cumulative distribution functions (CDFs) for spring wheat and maize yields. The nonparametric Kolmogorov–Smirnov (K–S) test was also conducted to determine if the projection period CDFs differed significantly from the baseline CDF [54,20].

#### Adaptation measures

Cultivars with altered phenology, fertilizer N application rates and planting dates were simulated based on sensitivity analyses in order to explore adaptation measures to climate changes on crop yield and nitrate leaching, under baseline and future climate scenarios for spring wheat at Swift Current and maize at Woodslee. Therefore, the CSM- CERES- Wheat and -Maize models were run 7050 times for adaptation measures analysis at both locations (Table C-b in S1 File). The management practice scenarios were:

- Fertilizer N application rates were simulated (0–100 and 0–300 kg N ha^-1^) with 20 and 50 kg N ha^-1^ increments for spring wheat and maize, respectively.
- Planting dates were simulated using 10, 20, 30 days before/after the default planting date for spring wheat and by using 15, 30, 45 days before/after the default planting dates for maize.
- Spring wheat cultivars with long growing seasons were explored based on the genotype parameters of PHINT (interval between successive leaf tip appearances,°C.d) and P5 (grain filling phase (excluding lag) duration,°C.d).
- Maize cultivars with long growing seasons were developed based on two thermal time parameters of P1 (thermal time from seedling emergence to the end of the juvenile phase (degree days > 8°C)) and P5 (thermal time from silking to physiological maturity, (degree days > 8°C).

## Results

### Projected climate change under future scenarios

The comparison of historical and projected climate normals (temperature, precipitation, solar radiation) and CO_2_ concentration at Swift Current, SK and Woodslee, ON is shown in Table 1. The corresponding changes in monthly normals are shown in Figures A and B in S1 File. Compared to the baseline scenario, the projected changes for annual precipitation under RCP4.5 2050s, RCP4.5 2080s, RCP8.5 2050s and RCP8.5 2080s scenarios increased by 5.4, 10.7, 14.1, 21.3% at Swift Current and by 3.0, 2.3, 16.1, 11.4% at Woodslee (Table 1). The predicted growing season rainfall from May to August for wheat and from May to October for maize did not differ from the baseline scenario under RCP4.5 2050s and RCP4.5 2080s conditions, but it increased by 11.9, 10.5% at Swift Current and by 14.5, 4.6% at Woodslee under RCP8.5 2050s and RCP8.5 2080s climate scenarios, respectively. The average CO_2_ concentrations increased by 152, 187, 232 and 461 ppm for RCP4.5 2050s, RCP4.5 2080s, RCP8.5 2050s and RCP8.5 2080s scenarios, respectively (Table 1).

**Table 1:** Climate normals under different climate scenarios at Swift Current, SK and Woodslee, ON.

Scenarios	Year	SRAD^1^	Tmax	Tmin	Tavg	Difference	CO_2_	Change^2^	P ^3^	Change	GSP	Change
		(MJ m^-2^ day^-1^)	(°C)	(°C)	(°C)	(°C)	(ppm)	%	(mm)	%	(mm)	%
**Swift Current**												
Baseline	1971–2000	13.7	9.6	-1.8	3.9		346		349		207	
RCP4.5	2050s	13.7	12.8	1.6	7.2	3.2	497	43.9	368	5.4	202	-2.7
	2080s	13.8	13.6	2.3	7.9	4.0	533	54.0	386	10.7	204	-1.7
RCP8.5	2050s	13.5	13.3	2.3	7.8	3.9	578	67.2	398	14.1	232	11.9
	2080s	13.4	15.8	5.0	10.4	6.5	807	133.5	423	21.3	229	10.5
**Woodslee**												
Baseline	1971–2000	13.7	13.8	4.6	9.2		346		845		473	
RCP4.5	2050s	13.8	16.6	7.4	12.0	2.8	497	43.9	871	3.0	458	-3.2
	2080s	14.0	17.4	8.0	12.7	3.5	533	54.0	865	2.3	470	-0.7
RCP8.5	2050s	13.8	17.3	8.0	12.6	3.4	578	67.2	981	16.1	541	14.5
	2080s	13.7	19.5	10.3	14.9	5.7	807	133.5	942	11.4	495	4.6

^1^ SRAD solar radiation, Tavg average temperature, P precipitation, GSP growing season precipitation.

^2^ Change % = (scenario-baseline)/baseline*100.

^3^ The SRAD, Tavg, CO_2_ and P represent average annual data of climate normals over the corresponding periods. The Tmin and Tmax are calculated based on average yearly minimum and maximum temperatures, respectively.

### Climate change impacts on crop yield

Compared with the baseline scenarios, the average number of cold days (<4°C) decreased by about 6–13 days and hot days (>30°C) increased by more than 20 days during the spring wheat season under future RCP scenarios at Swift Current (Table 2). The average number of cold days decreased by about 10–16 days and hot days increased by more than 30 days during the maize growing season at Woodslee under future RCP scenarios (Table 2). Under future climate change scenarios, the predicted anthesis dates were 7–10 days and 10–16 days earlier than the baseline (68 and 71 days after planting) for spring wheat and maize, respectively (Table 2). Similarly, the predicted maturity dates were 9–18 days and 37–56 days earlier than the baseline (100 and 155 days after planting) for spring wheat and maize, respectively.

**Table 2:** The simulated average hot and cold days in the growing season, anthesis and maturity dates for spring wheat at Swift Current, SK and maize at Woodslee, ON under different climate scenarios.

Scenarios	Year	Planting	Anthesis	Maturity	Average number	Average number
		date (day of year)	date (day of year)	date (day of year)	cold days^1^ (d)	hot days (d)
**Swift Current**						
Baseline	1971–2000	130	198	227	16.2	15.7
RCP4.5	2050s	130	191	216	9.8	39.7
	2080s	130	190	214	7.3	39.4
RCP8.5	2050s	130	191	214	7.2	41.6
	2080s	130	188	209	3.0	65.0
**Woodslee**						
Baseline	1971–2000	145	216	300	16.9	19.8
RCP4.5	2050s	145	206	263	6.9	51.4
	2080s	145	206	258	4.4	63.0
RCP8.5	2050s	145	205	257	4.3	68.3
	2080s	145	200	244	0.6	106.2

^1^ Cold days: daily minimum temperature ≤ 4°C

Hot days: daily maximum temperature ≥ 30°C.

**Fig 1:**
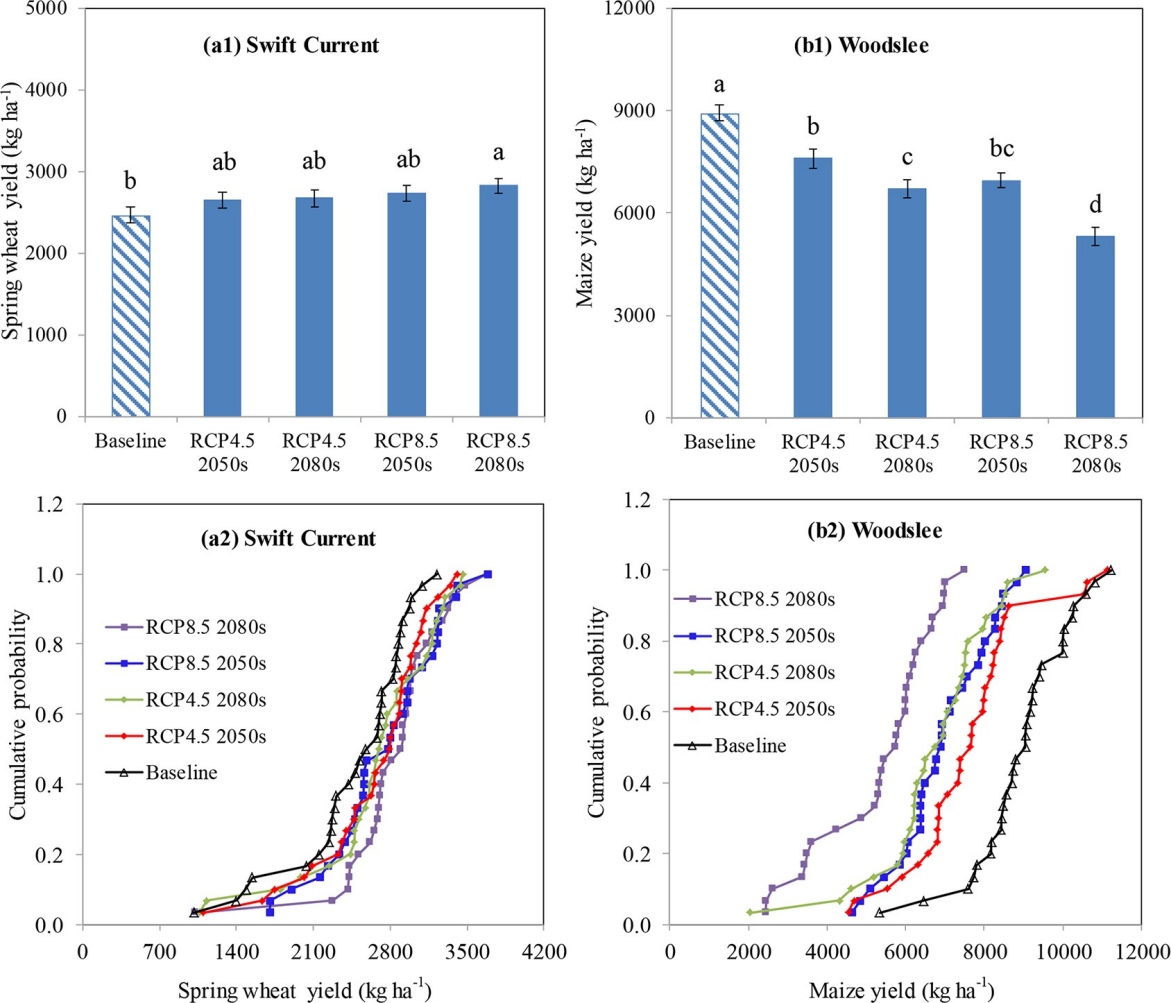
Effects of climate change scenarios on spring wheat (a1, a2) at Swift Current, SK and on maize yields (b1, b2) at Woodslee, ON. The lowercase letters represent statistical differences between different climate scenarios at the 0.05 level. Bars are standard deviations (n = 4).

The changes in cumulative distribution functions (CDFs) for the projection years were compared with the baseline for RCP4.5 2050s, RCP4.5 2080s, RCP8.5 2050s and RCP8.5 2080s to analyze the effects of climate change factors on wheat and maize growth yields (Fig 1, Figures B and C in S1 File.). At Swift Current, compared to baseline scenarios, the wheat yield increased by 8, 8, 11 and 15% under RCP4.5 2050s, RCP4.5 2080s, RCP8.5 2050s and RCP8.5 2080s, respectively (Fig 1A1). Projected increases in CO_2_ concentration and precipitation significantly (*p*<0.05) increased wheat yield. However, projected temperature changes significantly decreased wheat yields with substantial differences were found among projected scenarios as illustrated by the lowest yields associated with RCP8.5 for the 2080’s period (Figure C-a1, c1 in S1 File). With increasing CO_2_ concentrations alone, the yield increased by 8, 11, 12 and 16% for RCP4.5 2050s, RCP4.5 2080s, RCP8.5 2050s and RCP8.5 2080s, respectively (Figure C-a1 in S1 File). With temperatures increasing by 3.2, 4.0, 3.9, 6.5°C for RCP4.5 2050s, RCP4.5 2080s, RCP8.5 2050s and RCP8.5 2080s, the yield decreased significantly by 10, 15, 13 and 29% (Figure C-b1 in S1 File). The increases in precipitation resulted in a significant increase in wheat yields by about 10% (Figure C-c1 in S1 File). At Woodslee, maize yields decreased by 15, 25, 22 and 41% under RCP4.5 2050s, RCP4.5 2080s, RCP8.5 2050s and RCP8.5 2080s, respectively (Fig 1A2). Projected CO_2_ concentrations and precipitation did not have a notable impact on maize yield at Woodslee (Figure D-a1, c1 in S1 File). However, increased temperature significantly decreased maize yield by 18, 25, 26 and 44% under RCP4.5 2050s, RCP4.5 2080s, RCP8.5 2050s and RCP8.5 2080s, respectively (Figure D-b1 in S1 File).

The Kolmogorov-Smirnov (K-S) test for comparison of the CDFs of wheat and maize yields between climate change and baseline scenarios showed that the CDFs are significantly different from baseline yields using the D statistic (Table E in S1 File). The results showed that RCP8.5 2080s was the only scenario which had a significant D statistic for spring wheat yields (D = 0.37), whereas all the climate scenarios significantly affected maize yields (D ranged from 0.60–0.93) compared to the baseline scenarios (Fig 1, Table E in S1 File). With elevating CO_2_ concentration alone, all climate scenarios except RCP4.5 2050s had significant D statistics of 0.37–0.40 for spring wheat, but they did not significantly impact the CDFs of maize (i.e., D ranged from 0.2–0.3). Increased temperature significantly impacted the CDFs of spring wheat yields for RCP4.5 2080s and RCP8.5 2080s scenarios while temperature significantly impacted all the CDFs for maize yields under all four scenarios (Figures C and D, and Table E in S1 File).

### Climate change impacts on soil water balance

With increasing CO_2_ alone, evapotranspiration (ET) decreased at both locations under four climate scenarios (Table 3). The mechanisms responsible for these decreases differed between the two sites a only evaporation (ES) decreased at Swift Current whereas only transpiration (EP) decreased at Woodslee (Table 3). Based upon temperature increases alone, ET increased by an average of 7–12% at Swift Current, mainly due to the two-fold increase in ES values. ET also increased by from 7–13% at Woodslee which was due to the 14–20% increase in EP (Table 3). When all factors were combined, ET increased significantly at both Swift Current and Woodslee under future RCP climate scenarios.

**Table 3:** Simulated soil water balance components for spring wheat at Swift Current, SK and maize at Woodslee, ON under different climate scenarios.

		Swift Current						Woodslee					
Scenarios	Year	ET^1^	EP	ES	Drain	AW	WUE	ET	EP	ES	Drain	AW	WUE
		(mm)					(kg m^-3^)	(mm)					(kg m^-3^)
Baseline	1971–2000	304	147	65	46	249	1.04	612	371	166	207	361	1.69
		**CO**_**2**_											
RCP4.5	2050s	302	149	55	48	251	1.14	607	364	167	211	366	1.75
	2080s	302	150	52	48	252	1.16	605	362	167	213	367	1.77
RCP8.5	2050s	302	150	50	48	252	1.18	604	360	168	214	369	1.78
	2080s	300	151	39	50	256	1.24	595	350	168	223	376	1.85
		**Temperature**											
RCP4.5	2050s	324	136	109	27	228	0.91	656	423	147	163	325	1.31
	2080s	330	136	121	21	214	0.84	666	433	145	154	317	1.18
RCP8.5	2050s	327	136	115	24	213	0.87	669	436	146	151	314	1.15
	2080s	339	123	154	12	193	0.68	689	445	160	131	294	0.86
		**Precipitation**											
RCP4.5	2050s	320	162	49	49	341	1.11	621	375	167	220	351	1.69
	2080s	329	162	54	56	361	1.10	620	377	164	218	342	1.71
RCP8.5	2050s	333	164	58	64	367	1.09	624	373	171	317	379	1.66
	2080s	328	162	42	89	354	1.10	615	372	166	292	378	1.70
		**All factors combined**											
RCP4.5	2050s	325	160	67	44	246	1.07	661	426	147	182	322	1.34
	2080s	340	162	76	44	255	1.04	667	432	148	172	298	1.16
RCP8.5	2050s	337	160	77	59	263	1.07	678	438	153	264	335	1.19
	2080s	344	169	70	74	251	1.10	685	449	154	225	321	0.90

^1^ ET evapotranspiration, EP transpiration, ES evaporation, Drain cumulative drainage water, AW available soil water at maturity, WUE water use efficiency.

Annual soil cumulative drainage and available soil water (AW) at maturity did not change with CO_2_, but it did decrease with temperature and increase with precipitation under future climate scenarios at both locations (Table 3). When all factors were combined, significant increases in annual cumulative drainage were found under RCP8.5 2050s and RCP8.5 2080s scenarios compared with baseline scenarios, which were primarily due to the higher annual precipitation (Table 3).

Comparing the present and future scenarios, the average soil water use efficiency (WUE, as defined by yield per unit of actual evapotranspiration) increased with increasing CO_2_ concentrations due to the decreased ET. In contrast, the WUE decreased with temperature due to the predicted decrease in yields. At Woodslee, when all factors were combined, WUE decreased significantly under future RCP climate scenarios because of the significant decreases in maize yields.

### Climate change impacts on soil mineral and nitrate leaching

**Fig 2:**
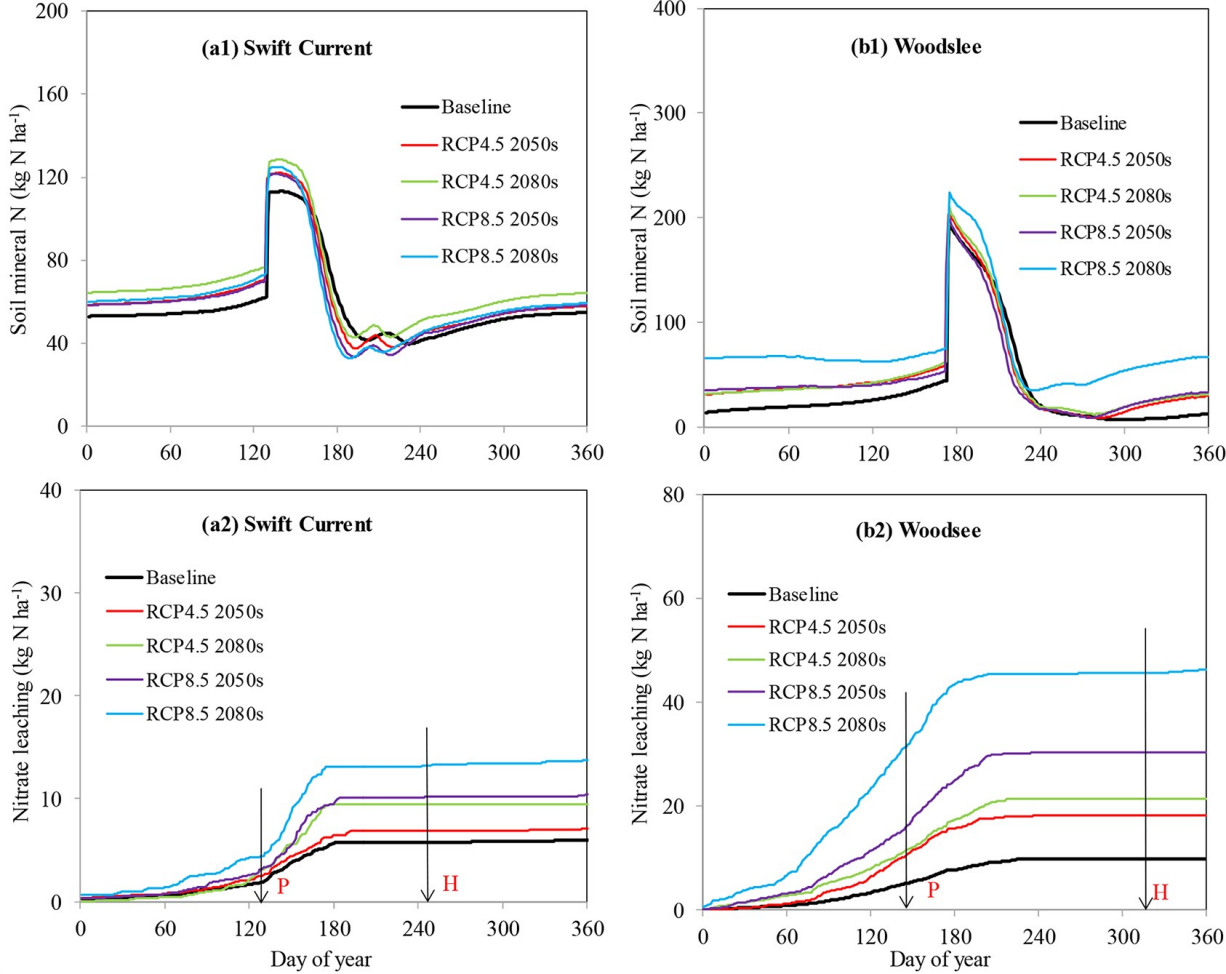
Effects of climate change on dynamics of soil mineral N and cumulative nitrate leaching in the spring wheat field (a1, a2) at Swift Current, SK and the maize field (b1, b2) at Woodslee, ON. Vertical lines with P and H in Fig a2 and b2 represent planting and harvest dates of spring wheat and maize, respectively.

Compared to baseline scenarios, the average soil mineral N increased by 6, 15, 3, 5% at Swift Current and by 28, 32, 26, 101% at Woodslee under RCP4.5 2050s, RCP4.5 2080s, RCP8.5 2050s and RCP8.5 2080s scenarios, respectively (Fig 2, Figures E and F in S1 File). Soil mineral N was significantly affected by temperature (Figures E-b1 and F-b1 in S1 File). In general, future elevated temperatures would significantly increase soil mineral N at both locations under future climate scenarios compared to the baseline scenario because high temperature resulted in higher residual N concentrations in the soil due to a lower yield and grain N uptake (Figures C and D in S1 File). Increased CO_2_ and precipitation decreased soil mineral N by about 21% and 27%, at Swift Current (Figure E-a1, c1 in S1 File), because both of these factors increased wheat yields and N uptake of soil mineral N (Figures C and D in S1 File). The reason for small increases in soil mineral N when all factors were combined at Swift Current was that the increased soil mineral N due to temperature increases was offset by the decreased soil mineral N resulting from elevated CO_2_ concentrations (i.e. increased yields and N uptake) and increasing precipitation (i.e. greater leaching) (Figure C-a4 in S1 File).

Nitrate leaching increased dramatically with time from the beginning of the year to late June, and then leveled off from July to the end of the year at both locations (Fig 2). There was more nitrate leaching during the growing seasons at Swift Current and during the non-growing seasons at Woodslee (Figure G in S1 File). Soil nitrate leaching was about 6 kg N ha^-1^ at Swift Current, and 11 kg N ha^-1^ at Woodslee under the baseline scenario. Annual nitrate leaching increased by 1.1, 3.5, 4.4 and 7.7 kg N ha^-1^ (19, 57, 73, 129%) at Swift Current and increased by 8, 12, 21 and 37 kg N ha^-1^ (84, 117, 208, 317%) at Woodslee under RCP4.5 2050s, RCP4.5 2080s, RCP8.5 2050s and RCP8.5 2080s climate scenarios, respectively (Fig 2A2 and 2B2). Increased CO_2_ did not impact soil nitrate leaching at either location (Figures E-a2 and F-a2 in S1 File). However, increased temperatures resulted in large increases in soil nitrate leaching by 56, 68, 100, 119% at Swift Current and by 104, 146, 165, 415% at Woodslee under RCP4.5 2050s, RCP4.5 2080s, RCP8.5 2050s and RCP8.5 2080s climate scenarios, respectively (Figures E-b2 and F-b2 in S1 File). Increased precipitation only significantly impacted nitrate leaching under RCP8.5 2050s by 22% and under RCP8.5 2080s by 65% at Swift Current whereas the corresponding increases were 80% for RCP8.5 2050s and 51% for RCP8.5 2080s at Woodslee, (Figures E-c2 and F-c2 in S1 File).

### Adaptation measures to climate change

#### Optimum fertilizer N rate

**Fig 3:**
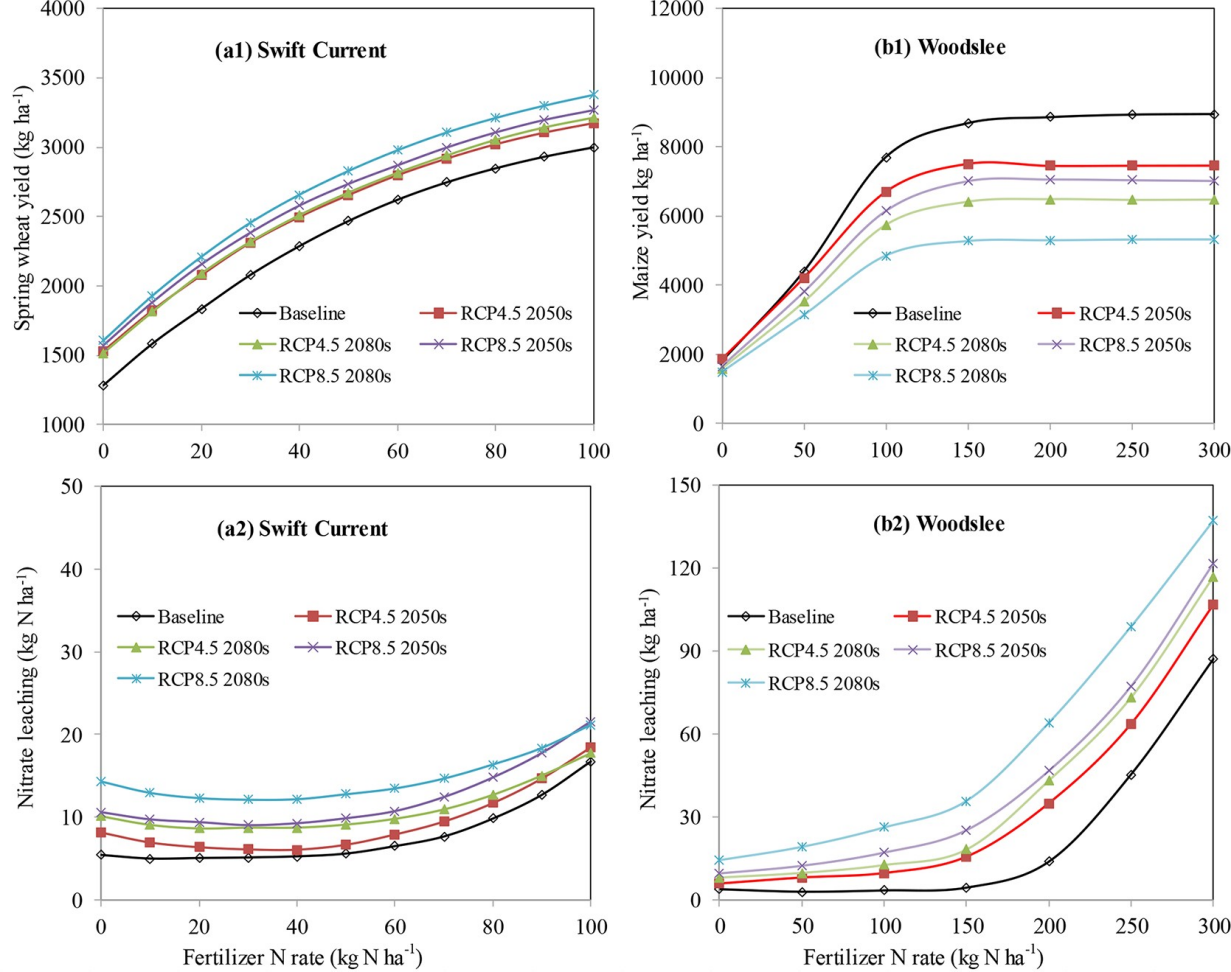
Sensitivity analysis of grain yields and soil nitrate leaching to fertilizer N rates at Swift Current, SK (a1, a2) and Woodslee, ON (b1, b2) under baseline and future climate scenarios.

The simulated wheat and maize yields showed the law of diminishing returns with fertilizer N rate (FNR) (Fig 3A1 and 3A2). At Swift Current, when the FNR was changed from 0 to 100 kg N ha^-1^, under the baseline and future climate scenarios (RCP4.5 2050s, RCP4.5 2080s, RCP8.5 2050s, and RCP8.5 2080s), wheat yield increased from 1283–1606 kg ha^-1^ to 2998–3376 kg ha^-1^ (Fig 3A1). Average N leaching was 8–13 kg N ha^-1^ when the FNR increased from 0 to 50 kg N ha^-1^ while it increased to 16–21 kg N ha^-1^ (104%) when the FNR increased from 50 to 100 kg N ha^-1^ (Fig 3B1). The results indicated that the baseline fertilizer N rate of 50 kg N ha^-1^ was insufficient to obtain high wheat yields and optimal yields were achieved at fertilizer N rates between 80 to 100 kg N ha^-1^ (Fig 3A1) although these rates may result in an additional 5–8 kg N ha^-1^ nitrate leaching losses (Fig 3B1).

At Woodslee, maize yields dramatically increased with an increased N application rate up to 150 kg N ha^-1^, and maize yields levelled off when the FNR increased from 150 to 300 kg N ha^-1^ for all scenarios (Fig 3A2). Based on our simulation (Fig 3), the maximum maize yield of 7500 kg ha^-1^ could be obtained at 150 kg N ha^-1^ under future RCP scenarios, while the maximum predicted maize yield of >9000 kg ha^-1^ could be achieved with 180–200 kg N ha^-1^ under baseline scenarios. On the other hand, simulated nitrate leaching increased exponentially at the higher N fertilizer rates of 150–300 kg N ha^-1^ because the crops could not utilize surplus fertilizer N in the soil (Fig 3B2). This illustrated that a FNR of 150 kg N ha^-1^ is a reasonable fertilizer N application rate to achieve a balance between high maize yields comparatively low nitrate leaching estimates for both current and future climate conditions (Fig 3B2).

#### Adjustment of planting date

**Fig 4:**
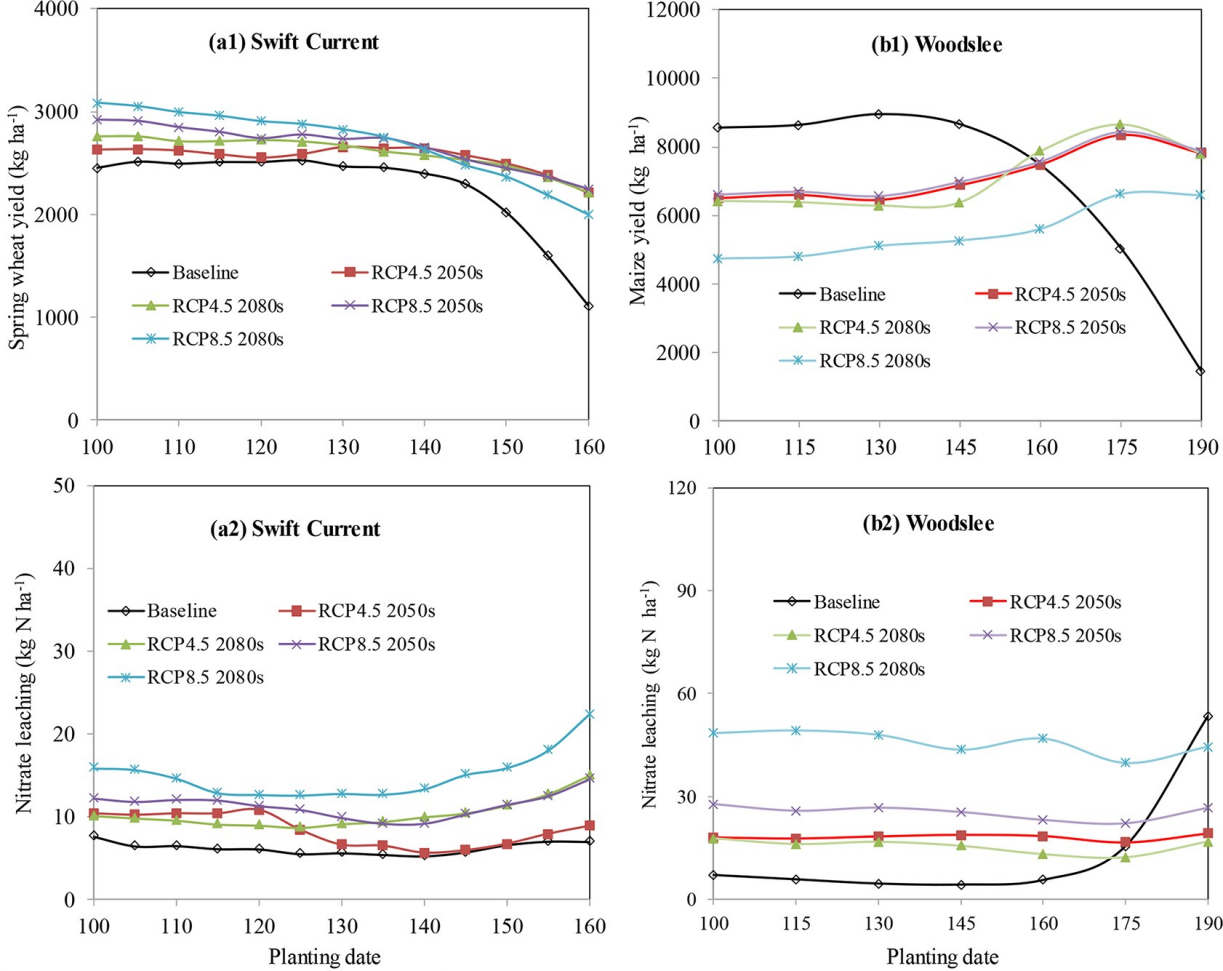
Sensitivity analysis of grain yields and soil nitrate leaching to planting dates at Swift Current, SK (a1, a2) and Woodslee, ON (b1, b2) under baseline and future climate scenarios.

Planting date (PD) affected crop yields differently at the two locations. At Swift Current, the wheat yield decreased and the nitrate leaching increased (Fig 4A1 and 4B1) when the PD was delayed. Wheat yield increased significantly when the PD was set to 15–20 days earlier than the baseline scenario, day 130, for the RCP8.5 2050s and RCP8.5 2080s scenarios (Fig 4A1). At Woodslee, the planting dates for the high yields were between days 130–140 for baseline scenarios, and between 170–180 for future climate scenarios, respectively (Fig 4A2). Delayed planting had an advantage for maize growth in the critical growth stage, as precipitation would increase significantly in August (Figure B-c in S1 File, Fig 4). Soil nitrate leaching significantly increased by delaying PD for the baseline scenario due to low crop N uptake, whereas it did not have a significant impact on nitrate leaching under future climate conditions.

#### Development of new cultivars

**Fig 5:**
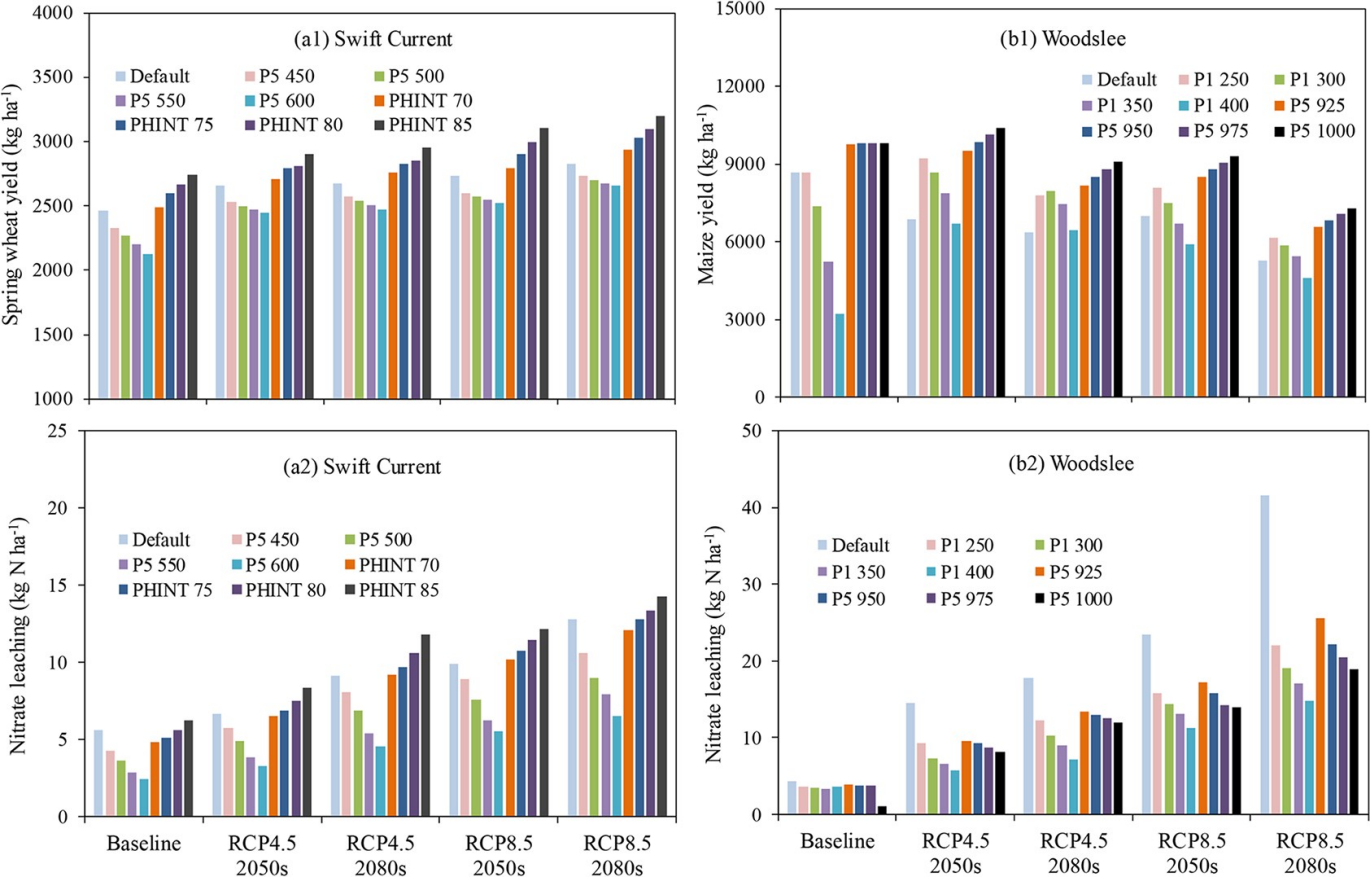
Sensitivity of yields and soil nitrate leaching to cultivar parameters under baseline and future climate scenarios at Swift Current, SK (a1, a2) and Woodslee, ON (b1, b2); Wheat cultivar PHINT represents interval between successive leaf tip appearances (°C.d) and P5 is grain filling (excluding lag) phase duration (°C.d). Maize cultivar P1 represents thermal time from seedling emergence to the end of the juvenile phase (degree days > 8°C) and P5 represents thermal time from silking to physiological maturity (degree days > 8°C).

Sensitivity analysis for selected wheat cultivar parameters of PHINT and P5, and maize cultivar parameters of P1 and P5 were carried out in order to recommend a new cultivar with longer growing season to adapt to future RCP climate conditions. At Swift Current, Changing P5 parameter from 400 (default) to 600 degree days did not improve wheat yield (Fig 5A1). However, the wheat yield significantly increased with the increases of PHINT parameter. For example, under RCP8.5 2080s scenario, wheat yield increased from 2828 kg ha^-1^ (PHINT at default value of 65) to 3196 kg ha^-1^ (PHINT = 85 degree days) (Fig 5A1), meanwhile soil nitrate leaching increased slightly (12.8 to 14.2 kg N ha^-1^) (Fig 5A2), indicating that PHINT is a key parameter for adapting to climate change. Soil nitrate leaching fell below 15 kg N ha^-1^ across all scenarios (Fig 5A2).

At Woodslee, maize yields increased from 6883 kg ha^-1^ with P1 parameter at default (212) to 9218 kg ha^-1^ with P1 at 250 degree days > 8 ^o^C (Fig 5B1). Maize yield also increased significantly with the increases of P5 parameter for all future climate scenarios except RCP8.5 2080s scenario. For example, under RCP4.5 2050s scenario, maize yield increased from 6883 to 9860 kg ha^-1^ when the P5 parameter changed from 890 (default) to 950 growing degree days (Fig 5B1) meanwhile soil nitrate leaching decreased from 15.6 to 9.3 kg N ha^-1^ under this scenario (Fig 5B2). RCP8.5 2080s scenario showed the lowest yields with highest soil nitrate leaching among all scenarios (Fig 5A2 and 5B2). There was a trend for decreasing soil nitrate leaching when maize yields increased because new maize cultivars with high growing degree days could obtain greater yields and greater N uptake which would reduce surplus fertilizer N remaining in the soil at the end of the growing season (Fig 5A2 and 5B2).

## Discussion

### Crop yield, soil water and nitrate leaching under future climate

In our study, climate change had a negative effect on maize yields and a positive effect on wheat yields without adaptation measures. Increased CO_2_ resulted in a significant increase in spring wheat yields but less than 5% increase in maize yields because of the growth characteristics of the different **c**rop types (e.g., wheat is a C3 crop and maize is a C4 crop). Hatfield et al. [55] concluded that the doubling of CO_2_ would result in about a 30% increase in C3 crop yields, compared to a less than 10% increase for C4 crops. Similar results were reported by Thomson et al. [56] and Ko et al. [57], where the increase in global mean temperature had a negative effect on crop yields, which was partially offset by the positive impact of increasing CO_2_ and precipitation. In addition, Ben-Asher et al. [58] found that photosynthetic rate declined for each 1°C increase in temperatures above 30°C. In the CERES-Maize, and -Wheat models, the rate of development is governed by thermal time or growing degree-days (GDD), which is computed based on the daily maximum and minimum temperatures and base temperature for root growth [11]. For example, the GDD from maize germination to emergence (P9) is calculated from seed depth (SDEPTH, cm) by P9 = 45.0 + GDDE × SDEPTH, where GDDE is an ecotype parameter that GDD per cm seed depth required for emergence [59].

The ET decreased with increasing CO_2_ alone at both locations under four climate scenarios. The reason for these differences is that elevated CO_2_ can lead to a decrease in stomatal conductance which would result in lower ET values and increased leaf temperatures [60]. In DSSAT-CERES modules, photosynthesis of plant leaves was computed hourly using the asymptotic exponential response equation, where quantum efficiency and light-saturated photosynthesis rate variables were dependent on CO_2_ and temperature [11,61]. In addition, our results showed that the ET increased with increasing temperatures which were in agreement with Wang et al. [20].

More nitrate leaching occurred during the growing seasons at Swift Current and the non-growing seasons at Woodslee. These results were due to the differences in growing season lengths and timing (early May to late August for Swift Current and late May to early November for Woodslee) as well as the differences in quantities and patterns of precipitation between these two locations. At Swift Current, only about 150 mm (<40%) of precipitation occurred in non-growing season, but for Woodslee, more than 400 mm (>55%) of precipitation occurred in the non-growing season. Under future climate scenarios, nitrate leaching increased significantly compared to the baseline scenario at Woodslee which was mainly due to the future high temperature stress and increased precipitations that resulted in low crop N removal and increased drainage [55].

### Adjustments of N application rate, planting date and cultivars

Fertilizer N rate will be expected to change under future climate to optimize crop yield due to changes in precipitation patterns, temperature and extreme weather events [19,20]. Our results indicated that the optimal spring wheat yields were achieved at higher fertilizer N rates (80 to 100 kg N ha^-1^) compared to the insufficient fertilizer N rate under both baseline and future climate conditions [49]. At Woodslee, simulated nitrate leaching increased significantly when fertilizer N rates were greater than 150 kg N ha^-1^ as maize yields did not respond to additional N fertilizer inputs above this rate and there would be surplus fertilizer N in the soil which could be lost by leaching. Therefore, the 150 kg N ha^-1^fertilizer N rate is recommended to apply for obtaining high maize yields with comparatively low nitrate leaching for both baseline and future climate scenarios.

Wheat yields increased significantly when PD was set to earlier than the baseline scenario at Swift Current under RCP8.5 scenarios. At this location, Bootsma and De Jong [62] estimated the ideal seeding date of spring wheat using selected environmental criteria including (a) snow cover<10 mm for the day; (b) Daily precipitation<2.5 mm; (c) 0.75Tmax+0.25Tmin>7°C; (d) The SW1 (soil water at the top 5% of the soil profile) < 90% Available Water Holding Capacity (AWHC); (e) SW2 (soil water at the next 7.5% of the profile) < 0.95 AWHC. The study concluded that the wheat seeding date can be 10 to 15 days earlier than the current planting date at Swift Current. Gan et al. [63] used a 4-year seeding date trial to determine that seeding 10 to 12 days earlier than baseline could increase grain yields of spring wheat near Swift Current. At Woodslee, late planting of maize in the baseline scenario had a negative effect on maize yield because maize cannot reach maturity with a reduced number of growing degree days and insufficient heat units. However, for future climate conditions, higher temperature can ensure that maize will get enough heat to mature. In addition, early planting under future climate scenarios could result in water stress for maize since the precipitation would on average, decrease significantly and the temperature would increase by 4°C in July compared to the baseline scenario. In contrast, delayed planting would be beneficial to maize growth in the critical growth stage because of the increased precipitation in August. In addition, soil nitrate leaching significantly increased when the PD was delayed for the baseline scenario due to low crop N removal, but it did not have a significant impact on nitrate leaching under future climate conditions. Our results were in agreement with Wang et al. [20], who reported that maize yields would increase from delayed planting dates under future climate scenarios in Iowa (similar climate to southwestern Ontario). In contrast, Babel and Turyatunga [64] illustrated that the maximum maize yields could be obtained by early planting under SRES scenarios, which could be attributed to the differences in local climate patterns (e.g. distributions of precipitation), crop management practices (e.g. tillage, fertilizer, cultivars), and IPCC scenarios (e.g. SRES, RCPs).

Compared to the baseline cultivars, the wheat yield of new cultivar increased with the increased PHINT whereas maize yield increased with the parameters of P1 and P5. Thus development of new cultivars indicated that long growing season with higher thermal degree days are required to adapting to climate change in the future. This result was consistent with previous modelling studies using the RCP or SRES scenarios for winter wheat and maize. Future increased temperature conditions were found to require longer growing season cultivars to reach crop maturity for obtaining high yields and reduce environmental risks [19,41].

### Uncertainties and limitations

Uncertainties in projections of climate change impacts on agro-ecosystems are due to several factors including differences in climate and crop models, emission scenarios and down-scaling techniques [65,66]. Future greenhouse gas emission estimates are also strongly affected by socio-economic development and political strategies which contribute to another source of uncertainty. There are also some uncertainties generated from the inherent model structure and the model calibration, parameterization and evaluation [61,65].

In this study, climate scenarios from the CanRCM4 model was used, thus the uncertainty associated with climate scenarios could be large based on the previous comparisons between global climate models or regional climate models [44,67,68], However, this could be improved in the future by driving the DSSAT model with a range of future climate scenarios from various climate models. The DSSAT model was calibrated and evaluated using long-term field experiments for spring wheat and maize. However, the lack of measured values for nitrate leaching at Swift Current and insufficient measurements of soil mineral and nitrate leaching at Woodslee contributed to the uncertainty in the model performance. In addition, initial soil parameters were set up for each scenario before the first year simulation, but certain soil physical properties such as hydraulic conductivity and water holding capacity were kept constant even though they may change under different climate conditions and management practices (e.g., fertilization, rotation, tillage) which could further impact crop yield and soil process. High crop production via crop residue inputs may increase carbon returns to soil but higher temperatures may also accelerate soil organic carbon decomposition [69]. Thus the net change in soil organic carbon levels can have a positive or negative impact on soil hydraulic properties which are not all captured in the current DSSAT model.

## Conclusions

Climate change impacts on spring wheat and maize yields, soil water balance and soil nitrogen dynamics were determined using the DSSAT model. Simulation results indicated that the spring wheat yields increased and maize yields decreased under future RCP scenarios, respectively. Increased CO_2_ and precipitation had a positive impact on crop yields, but temperature showed significant negative effects on spring wheat and maize yields. In this study, ET increased for both locations under future RCP scenarios. Significant increases in cumulative drainage water losses were found under RCP8.5 2050s and RCP8.5 2080s scenarios compared to baseline scenarios as a result of the higher annual precipitation. More nitrate leaching occurred during the growing season at Swift Current whereas it was during non-growing season at Woodslee. Annual average nitrate leaching increased significantly at Woodslee under future climate scenarios mainly due to the future high temperature stress and increased precipitations that resulted in low crop N removal and increased drainage.

Adaptation measures of increasing fertilizer N rate from 50 (default) to 80–100 kg N ha^-1^ could significantly increase wheat yield, however nitrate leaching would increase by 5–8 kg N ha^-1^ at Swift Current. The fertilizer N rate of 150 kg N ha^-1^ is required to obtain high maize yields with low nitrate leaching under future climate conditions at Woodslee. Early planting could contribute to a higher wheat yield under baseline and future RCP8.5 scenarios at Swift Current, whereas late planting dates could reduce the negative impacts of climate change on maize production at Woodslee. Developing longer growing-season wheat and maize cultivars should be recommended as an adaptive management strategy to optimize wheat and maize yields production meanwhile minimize soil nitrate leaching under changing climatic conditions.

## Supporting information

S1 File The supporting information (SI) is provided in one single file. **Materials and methods**. Bias correction for climate scenarios; DSSAT model inputs and calibration. **Table A**. Summary of input parameters for DSSAT CSM-Wheat and Maize simulation. **Table B**. The calibrated cultivar coefficients for spring wheat at Swift Current and maize at Woodslee. **Table C**. Summary of simulation runs using DSSAT v4.6. **Table D**. Statistical evaluation of DSSAT simulation performance. **Table E**. K-S test of cumulative distribution functions (CDFs) for spring wheat at Swift Current and maize at Woodslee. **Figure A**. Monthly climate normals under different climate scenarios at Swift Current. **Figure B**. Monthly climate normals under different climate scenarios at Woodslee. **Figure C**. Effects of CO_2_, temperature and precipitation on spring wheat yield (a1-a2) under different climate scenarios at Swift Current. **Figure D**. Effects of CO_2_, temperature and precipitation on maize yield (a1-a2) under different climate scenarios at Woodslee. **Figure E**. Effects of CO_2_, temperature and precipitation on soil mineral N and nitrate leaching (a1-a2) under different climate scenarios at Swift Current. **Figure F**. Effects of CO_2_, temperature and precipitation on soil mineral N and nitrate leaching (a1-a2) under different climate scenarios at Woodslee. **Figure G**. Effects of climate change on soil N leaching during growing and non-growing seasons.
(DOCX)
