# Supplementary material for: Climate change impacts on crop yield, soil water balance and nitrate leaching in the semiarid and humid regions of Canada
Source: PLoS One. 2018 Nov 16;13(11):e0207370. doi: 10.1371/journal.pone.0207370 (PMC6239327; doi:10.1371/journal.pone.0207370)
Supplement: S1 File — Materials and methods. Bias correction for climate scenarios; DSSAT model inputs and calibration. Table A. Summary of input parameters for DSSAT CSM-Wheat and Maize simulation. Table B. The calibrated cultivar coefficients for spring wheat at Swift Current and maize at Woodslee. Table C. Summary of simulation runs using DSSAT v4.6. Table D. Statistical evaluation of DSSAT simulation performance. Table E. K-S test of cumulative distribution functions (CDFs) for spring wheat at Swift Current and maize at Woodslee. Figure A. Monthly climate normals under different climate scenarios at Swift Current. Figure B. Monthly climate normals under different climate scenarios at Woodslee. Figure C. Effects of CO2, temperature and precipitation on spring wheat yield (a1-a2) under different climate scenarios at Swift Current. Figure D. Effects of CO2, temperature and precipitation on maize yield (a1-a2) under different climate scenarios at Woodslee. Figure E. Effects of CO2, temperature and precipitation on soil mineral N and nitrate leaching (a1-a2) under different climate scenarios at Swift Current. Figure F. Effects of CO2, temperature and precipitation on soil mineral N and nitrate leaching (a1-a2) under different climate scenarios at Woodslee. Figure G. Effects of climate change on soil N leaching during growing and non-growing seasons. (DOCX) [file pone.0207370.s001.docx]

**Supporting Information**

# Materials and methods

## Bias correction for climate scenarios

Biases are often found in the modelled data when they are compared with local climate observations. There are several reasons why these biases occur including systematic model errors and inaccuracies in parameterization, conceptualization and spatial averaging [1,2,3]. Methods have been developed to correct these biases in the RCM outputs. Simulations using climate change impact models such as hydrological and crop growth models have been found to have greater agreement with measured values when they use bias-corrected RCM data compared to uncorrected data [3,4]. The bias corrected modelled weather data can then be used as input for other crop-soil-water models as well as when simulating crop, nutrient and water responses to climate change scenarios [1,2,5].

Changes in the probability distribution of climate variables should be incorporated when the bias correction method is utilized in the future periods (i.e. 2041-2070 or 2071-2100) [1]. Future climate scenarios from the CanRCM4 model were bias-corrected with the equidistant Cumulative Distribution Function (CDF) matching method as described by Li et al. [6] and Qian et al. [1]. Observed station data of daily maximum and minimum air temperatures, precipitation and solar radiation for the historical period of 1971-2000 at Swift Current and Woodslee were obtained from Environment Canada and used for the purpose of bias correction.

## DSSAT model inputs and calibration

For the DSSAT application, the required input information included field management practices, daily weather inputs, soil profile data, cultivar characteristics, initial soil water content and inorganic N (NO_3_-N, NH_4_-N). Management practices included planting dates and density, fertilizer application rates and times, tillage types, irrigation and the amount and method of residue incorporation. The minimum weather inputs included daily maximum and minimum temperature (°C), daily solar radiation (MJ m^-2^) and daily precipitation (mm). The soil profile data included field capacity (m^3^ m^-3^), wilting point (m^3^ m^-3^), saturated water content (m^3^ m^-3^), soil bulk density (Mg m^‒3^), pH, organic C (kg C ha^-1^), silt content (%) and clay content (%).

The DSSAT model was calibrated and evaluated with the experimental data based on two field experiments. The first was a continuous long-term spring wheat study conducted from 1967 to 2005 at Swift Current, Saskatchewan, Canada. Initial soil organic carbon was 1.74% and conventional tillage was used in this experiment. The second experiment was a continuous long term maize study conducted from 1959 to present at Woodslee, Ontario, Canada. Initial soil organic carbon was 1.85%. Each year after harvest, maize residues were incorporated into the soil by mouldboard ploughing. Details on the experimental sites were described by Drury and Tan [7], Drury et al. [8], Liu et al. [9,10], and Li et al. [11]. The CSM-CERES-Wheat model in DSSAT was calibrated and evaluated using wheat yield, N uptake, soil water content and soil NO_3_-N in the 0-0.15 m from 1967 to 2005 at Swift Current and the CSM-CERES-Maize model was calibrated and evaluated using maize yields (1959-2012), above-ground biomass (2007-2012), soil mineral N (NO_3_^-^ + NH_4_^+^) in the 0-0.30 m (2007-2009), and soil nitrate leaching (1998-2000) at Woodslee (S4 Table). Detailed calibrated cultivar information can be found in the previous modelling studies [9,10,11]. Initial conditions, as well as soil and crop management data pertinent to this study were summarized in S1 Table. The calibrated parameters were based upon the previous studies of AC Barrie for wheat [11] and N58D1 for maize [10]. They are current popular cultivars used in study sites (Table B in S1 file). We then used them for both baseline and future climate scenarios to simulate the impact of climate change on spring wheat and maize yields, soil water balance and soil N dynamics.

Statistics of index of agreement (d), the mean error (E), and normalized root mean square error (nRMSE) were used to evaluate the DSSAT model’s performance [12,13]. Based on previous simulations, the results of d statistics showed good agreements between the simulated and measured data both at Swift Current (0.70≤d≤0.86) and Woodslee (0.81≤d≤0.96). Based on paired-t test on E values, there were statistically insignificant differences between the simulated and measured wheat yields, grain N uptake, soil water content, soil mineral N and soil NO_3_^-^N leaching. The nRMSE (<30%) values had acceptable agreements between the simulations and measurements excluding soil N which had a larger variation (nRMSE>30%) [9,10,11]. The overall results of the statistics evaluations had good agreements between the simulated and measured values (S4 Table).

# References

1. Qian B, De Jong R, Huffman T, Wang H, Yang JY. Projecting yield changes of spring wheat under future climate scenarios on the Canadian Prairies. Theor Appl Climatol. 2016; 123 (3-4): 651-669.
2. Christensen JH, Boberg F, Christensen OB, Lucas-Picher P. On the need for bias correction of regional climate change projections of temperature and precipitation. Geophys Res Lett. 2008; 35: L20709.
3. Teutschbein C, Seibert J. Bias correction of regional climate model simulations for hydrological climate-change impact studies: Review and evaluation of different methods. J Hydrol. 2012; 456–457: 12–29.
4. Oettli P, Sultan B, Baron C, Vrac M. Are regional climate models relevant for crop yield prediction in West Africa? Environ Res Lett. 2011; 6: 014008.
5. Ines AVM, Hansen JW, Robertson AW. Enhancing the utility of daily GCM rainfall for crop yield prediction. Int J Climatol. 2011; 31: 2168-2182.
6. Li H, Sheffield J, Wood EF. Bias correction of monthly precipitation and temperature fields from Intergovernmental Panel on Climate Change AR_4_ models using equidistant quantile matching. J Geophys Res. 2010; 115: D10101.
7. Drury CF, Tan CS. Long-term (35 years) effects of fertilizstion, rotation and weather on corn yields. Can J Plant Sci. 1995; 75: 355–362.
8. Drury C F, Reynolds W D, Tan C S, McLaughlin NB, Yang XM, Calder W, et al. Impacts of 49-51 years of fertilization and crop rotation on growing season nitrous oxide emissions, nitrogen uptake and corn yields. Can J Soil Sci. 2014; 94: 421-433.
9. Liu S, Yang JY, Drury CF, Liu HL, Reynolds WD. Simulating maize (*Zea mays* L.) growth and yield, soil nitrogen concentration, and soil water content for a long-term cropping experiment in Ontario, Canada. Can J Soil Sci. 2014; 94: 435‒452.
10. Liu HL, Yang JY, Drury CF, Reynolds WD, Tan CS, Bai YL, et al. Using the DSSAT-CERES-Maize model to simulate crop yield and nitrogen cycling in fields under long-term continuous maize production. Nutr Cycl Agroecosyst. 2011; 89: 313‒328.
11. Li ZT, Yang JY, Smith WN, Drury CF, Lemke RL, Grant B, et al. Simulation of long-term spring wheat yields, soil organic C, N and water dynamics using DSSAT-CSM in a semi-arid region of the Canadian prairies. Nutr Cycl Agroecosyst. 2015; 101: 401–419.
12. Yang JM, Yang JY, Liu S, Hoogenboom G. An evaluation of the statistical methods for testing the performance of crop models with observed data. Agric Syst. 2014; 127: 81–89.
13. Priesack E, Gayler S, Hartmann HP. The impact of crop growth sub-model choice on simulated water and nitrogen balances. Nutr Cycl Agroecosyst. 2006; 75: 1–13.

**Table A. Summary of input parameters for DSSAT CSM-Wheat and Maize simulation.**

|  | Input parameters | Swift Current | Woodslee |
| --- | --- | --- | --- |
| Crop management | Crop | Spring wheat | Maize |
|  | Cultivar | AC Barrie | N58D1 |
|  | Planting date (date) | 10-May | 25-May |
|  | Plant density (no. m^-2^) | 190 | 6 |
|  | Fertilizer N rate (kg N ha^-1^) | 50 | 150 |
|  | Tillage depth (m) | 0.1 | 0.2 |
| Soil input parameters | Soil profile depth (m) | 1.2 | 1.2 |
|  | Soil layers (cm) | 0-15, 15-30, 30-60, | 0-10, 10-20, 20-30, 30-40, |
|  |  | 60-90, 90-120 | 40-60, 60-120 |
|  | Bulk density (Mg m^‒3^) | 1.22 | 1.45 |
|  | Soil organic C (kg C ha^-1^) | 1.74 | 1.84 |
| Top soil | Field capacity (m^3^m^‒3^ ) | 0.268 | 0.341 |
| (0-0.20 m) | Wilting point (m^3^m^‒3^ ) | 0.117 | 0.118 |
|  | Saturated water content (m^3^m^‒3^ ) | 0.540 | 0.434 |
|  | Silt content (%) | 50.0 | 33.7 |
|  | Clay content (%) | 20.0 | 40.1 |
|  | pH | 6.8 | 6.0 |
| Initial conditions | Soil water content (m^3^m^‒3^ ) | 0.228 | 0.364 |
|  | Soil NH_4_ (kg N ha^-1^) | 6.9 | 4.8 |
|  | Soil NO_3_ (kg N ha^-1^) | 48.2 | 33.2 |
| Weather | Location | 50°170’N, 107°480’W | 42°13’N, 82°45’W |
|  | Elevation (m) | 756 | 186 |
|  | Daily weather variables | Solar radiation, Tmax, | Solar radiation, Tmax, |
|  |  | Tmin and precipitation | Tmin and precipitation |

**Table B. The calibrated cultivar coefficients for spring wheat at Swift Current and maize at Woodslee.**

| Cultivar | | Parameter descriptions | | | | | | | | Default | Calibrated^1^ |
| --- | --- | --- | --- | --- | --- | --- | --- | --- | --- | --- | --- |
| parameter | | |  |  |  |  |  |  |  |  |  |
| Spring wheat |  | |  |  |  |  |  |  |  | IB1500 | AC Barrie |
| P1V | Days, optimum vernalizing temperature, required for vernalization | | | | | | | | | 8 | 15 |
| P1D | Photoperiod response (% reduction in rate/10 h drop in pp) | | | | | | | | | 100 | 40 |
| P5 | Grain filling (excluding lag) phase duration (°C.d) | | | | | | | | | 320 | 400 |
| G1 | Kernel number per unit canopy weight at anthesis (#/g) | | | | | | | | | 23 | 16 |
| G2 | Standard kernel size under optimum conditions (mg) | | | | | | | | | 23 | 24 |
| G3 | Standard, non-stressed mature tiller weight (incl grain) (g dry weight) | | | | | | | | | 2.5 | 1.5 |
| PHINT | Interval between successive leaf tip appearances (degree days) | | | | | | | | | 86 | 65 |
| Maize |  | |  |  |  |  |  |  |  | IB10069 | N58 D1 |
| P1 | Thermal time from seedling emergence to the end of the juvenile phase ( degree days | | | | | | | | | 212.4 | 212.4 |
|  | > 8 °C) during which the plant is not responsive to changes in photoperiod | | | | | | | | |  |  |
| P2 | Extent to which development (expressed as days) is delayed for each hour | | | | | | | | | 0.52 | 0.52 |
|  | increase in photoperiod > the longest photoperiod 12.5 hours) | | | | | | | | |  |  |
| P5 | Thermal time from silking to physiological maturity ( degree days > 8 °C) | | | | | | | | | 792.8 | 890.4 |
| G2 | Maximum possible number of kernels per plant | | | | | | | | | 625 | 723 |
| G3 | Kernel filling rate during the linear grain filling stage and under optimum conditions (mg/day) | | | | | | | | | 6 | 6.9 |
| PHINT | Phylochron interval; the interval in thermal time (degree days) | | | | | | | | | 38.9 | 38.9 |

^1^ The cultivar coefficients of maize and wheat were calibrated by Liu et al. [53] and Li et al. [54], respectively.

**Table C.** **Summary of simulation runs using DSSAT v4.6.**

| **(a) Climate scenario analysis** | Baseline scenario | Climate scenarios | sub-scenarios | Simulation years | Runs |
| --- | --- | --- | --- | --- | --- |
| Swift Current | 1 | 4 | 4 | 30 | 510 |
| Woodslee | 1 | 4 | 4 | 30 | 510 |
| Total simulation runs |  |  |  |  | 1020 |
|  |  |  |  |  |  |
| **(b) Adaption analysis** | Baseline scenario | Climate scenarios | Parameter levels^1^ | Simulation years | Runs |
| Swift Current | 1 | 4 | 23 | 30 | 3450 |
| Woodslee | 1 | 4 | 24 | 30 | 3600 |
| Total simulation runs |  |  |  |  | 7050 |

^1^ Detailed parameter and levels are given in Table 2, including fertilizer N rate, planting date,

PHINT, P1 and P5.

**Table D.** **Statistical evaluation of DSSAT simulation performance.**

| Location | Item | Year | Measured^1^ | Simulated | Sample | E | nRMSE | d |
| --- | --- | --- | --- | --- | --- | --- | --- | --- |
|  |  |  |  |  | no. |  | % |  |
| Swift Current | Grain yield (kg ha^-1^) | 1967-2005 | 1635 | 1739 | 39 | 104 | 29.6 | 0.84 |
|  | Grain N uptake (kg N ha^-1^) | 1967-2005 | 43.4 | 52.3 | 38 | 8.9 | 36.2 | 0.79 |
|  | Soil water content (m^-3^ m^-3^) | 1967-2005 | 0.191 | 0.194 | 117 | 0.004 | 24.5 | 0.86 |
|  | Soil NO_3_-N (kg N ha^-1^) | 1967-2005 | 16.1 | 17.2 | 205 | 1.7 | 88.0 | 0.70 |
| Woodslee | Grain yield (kg ha^-1^) | 1959-2012 | 5069 | 5173 | 54 | 105 | 28.1 | 0.81 |
|  | Above-ground biomass (kg ha^-1^) | 2007-2012 | 7647 | 6571 | 41 | -1076 | 28.8 | 0.96 |
|  | Soil mineral N (mg kg^-1^) | 2007-2009 | 16.8 | 20.2 | 22 | 3.4 | 42.4 | 0.81 |
|  | N leaching (kg N ha^-1^) | 1998-2000 | 4.3 | 4.8 | 19 | 0.50 | 29.0 | 0.85 |

Liu et al., 2011, 2014; Li et al., 2015.

^1^ Measured and simulated represent average values over the corresponding periods.

**Table E.** **K-S test of cumulative distribution functions (CDFs) for spring wheat at Swift Current and maize at Woodslee.**

| Scenarios |  | D^1^ values | | | |
| --- | --- | --- | --- | --- | --- |
|  |  | CO_2_ effect | Temperature | Precipitation | All factors combined |
| Swift Current |  |  |  |  |  |
| RCP4.5 | 2050s | 0.33 | 0.27 | 0.33 | 0.23 |
|  | 2080s | 0.37^*2^ | 0.37^*^ | 0.27 | 0.23 |
| RCP8.5 | 2050s | 0.37^*^ | 0.30 | 0.30 | 0.30 |
|  | 2080s | 0.40^*^ | 0.47^*^ | 0.27 | 0.37^*^ |
| Woodslee |  |  |  |  |  |
| RCP4.5 | 2050s | 0.20 | 0.70^*^ | 0.17 | 0.60^*^ |
|  | 2080s | 0.20 | 0.77^*^ | 0.10 | 0.73^*^ |
| RCP8.5 | 2050s | 0.20 | 0.77^*^ | 0.10 | 0.63^*^ |
|  | 2080s | 0.30 | 0.93^*^ | 0.17 | 0.93^*^ |

^1^D value was generated from the Kolmogorov-Smirnov (K-S) test.

^2*^ represents significant difference compared with baseline at p < 0.05.

**Figure A. Monthly climate normals under different climate scenarios at Swift Current.**

**Figure B. Monthly climate normals under different climate scenarios at Woodslee.**

**Figure C. Effects of CO_2_, temperature and precipitation on spring wheat yield (a1-a2) under different climate scenarios at Swift Current.**

**Figure D. Effects of CO_2_, temperature and precipitation on maize yield (a1-a2) under different climate scenarios at Woodslee.**

**Figure E. Effects of CO_2_, temperature and precipitation on soil mineral N and nitrate leaching (a1-a2) under different climate scenarios at Swift Current.**

**Figure F. Effects of CO_2_, temperature and precipitation on soil mineral N and nitrate leaching (a1-a2) under different climate scenarios at Woodslee.**

**Figure G. Effects of climate change on soil N leaching during growing and non-growing seasons.**
